# Supplementary material for: The molecular interaction pattern of lenvatinib enables inhibition of wild-type or kinase-mutated FGFR2-driven cholangiocarcinoma
Source: Nat Commun. 2024 Feb 12;15:1287. doi: 10.1038/s41467-024-45247-6 (PMC10861557; doi:10.1038/s41467-024-45247-6)

THE MOLECULAR INTERACTION PATTERN OF LENVATINIB ENABLES INHIBITION OF WILD-TYPE OR KINASE-MUTATED FGFR2-DRIVEN CHOLANGIOCARCINOMA

Supplementary Methods, Results and Discussion

Supplementary Methods

Western Blot

Cells were lysed on ice with using RIPA buffer (EMD Millipore, Billerica, MA, USA) containing Proteinase and Phosphatase Inhibitor (Thermo Fisher Scientific, Schwerte, Germany) and phenylmethylsulfonyl fluoride (Sigma-Aldrich, Taufkirchen, Germany). Reducing NuPAGE LDS Sample Buffer (Thermo Fisher Scientific, Schwerte, Germany) and dithiothreitol (DTT) (Thermo Fisher Scientific) were added and proteins were denatured for 5 min at 95°C. Protein samples were loaded, separated by electrophoresis and transferred onto nitrocellulose membranes with a Trans-blot Turbo Transfer System (Bio-Rad, München, Germany). Membranes were blocked in TBS-T with 5% nonfat dry milk, followed by incubation with the primary antibody. Primary antibodies are specified in Supplementary Table 5. Membranes were incubated with the secondary mouse or rabbit antibody (both: Invitrogen by Thermo Fisher Scientific) in a concentration of 1:1000. Peroxidase activity was detected using Clarity Western ECL Substrate (Bio-Rad) and analyzed using X-ray cassettes with radiographic films (Thermo Fisher Scientific). To determine the relative protein abundance, densitometry of the total surface area of the respective bands was performed and normalized to the respective band of beta-actin or vinculin using ImageJ.

DigiWest Method[1] (see workflow in Supplementary Fig. 4)

Lysis and protein quantification. Cells were lysed on ice using 20-30 µl of 2x LDS Lysis Buffer (Life Technologies, Carlsbad, CA, USA), supplemented with a sample reducing agent (Thermo Fisher Scientific, Waltham, MA, USA), Protease and Phosphatase Inhibitor (Roche Diagnostics GmbH, Mannheim, Germany). Proteins were denatured at 95°C for 10 min before the lysates were transferred to QiaShredder tubes (Eppendorf, Hamburg, Germany). After centrifugation protein quantification was performed using in-gel staining. 1 µl of each original lysate was diluted in lysis buffer, and 10 µL were run in a NuPAGE 4-12% Bis-Tris precast gel (Thermo Fisher Scientific). All separated proteins were stained with BlueBandit (VWR, Darmstadt, Germany) for 1 h. After de-staining, detection was performed on a LI-COR (LI-COR, Bad Homburg, Germany) instrument. Quantification was performed using ImageStudio.

DigiWest procedure. For DigiWest, 10-12 µg of cellular protein were loaded on an SDS-polyacrylamide gel and size-separated using the commercial NuPAGE system (Life Technologies). Size-separated proteins were blotted onto a PVDF membrane and biotinylated on the membrane using NHS-PEG12-Biotin (50 µM) in PBST for 1 h. After drying of the membrane, the samples lanes were cut into 96 strips of 0.5 mm width using an automated cutting plotter (Silhouette America, West Orem, UT, USA) each corresponding to a defined molecular weight fraction. Each of the strips was placed in one well of a 96-well plate and 10 µl elution buffer (8 M urea, 1% Triton-X100 in 100 mM Tris-HCl pH 9.5) was added. The eluted proteins were diluted with 90 µl of dilution buffer (5% BSA in PBS, 0.02% sodium azide, 0.05% Tween-20) and each of the protein fractions was incubated with 1 distinct magnetic color-coded bead population (Luminex, Austin, USA) coated with neutravidin. The biotinylated proteins bind to the neutravidin beads such that each bead color represents proteins of one specific molecular weight fraction. All 96 protein loaded bead populations were mixed resulting in reconstitution of the original lane. Such a bead-mix was sufficient for about 150 individual antibody incubations. Aliquots of the DigiWest bead-mixes (about 1/200th per well) were added to 96-well plates containing 50 µl assay buffer. Blocking reagent for ELISA (Roche, Rotkreuz, Switzerland) supplemented with 0.2% milk powder, 0.05% Tween-20 and 0.02% sodium azide) and different diluted primary antibodies were added to the wells. A complete list of all antibodies used for DigiWest can be found in the Supplementary Data 1. After overnight incubation at 15°C in a shaker, the bead-mixes were washed twice with PBST and species-specific phycoerythrin-labeled secondary antibodies (Dianova, Hamburg, Germany) were added and incubated for 1 h at 23°C. Beads were washed twice prior to readout on a Luminex FlexMAP 3D.

For quantification of the antibody-specific signals, an Excel-based analysis tool was employed [1] that automatically identifies peaks of appropriate molecular weight and calculates the peak area (reported as accumulated fluorescence intensity = AFI). Signal intensity was normalized to the total amount of protein loaded onto one lane. For heatmap generation, data was median-centered and Log-2 transformed. HCL was performed using Pearson Correlation and complete linkage.

The full set of all included Digi West Experiments is shown in the Supplementary Data 1.

Sulforhodamine B (SRB) Assay

Cells (3.000/well) were seeded in duplicates in 24-well plates. After 24 hours, cells were treated with the indicated substances and concentrations. After treatment for seven days, cells were fixed for 30 minutes at 4°C with 10% trichloroacetic acid (Carl Roth, Karlsruhe, Germany), washed, and dried for 12 hours. The remaining cells were stained for 20 minutes with 0.4% SRB solution (Thermo Fisher Scientific, Schwerte, Germany), washed until the supernatant was colorless with 1% acetic acid, and dried again. The SRB solution was then discarded, and afterward, the cells were rinsed with 1% acetic acid and dried. Stained cells were solubilized in 10 mM TRIS per well for 10 minutes, and absorption of duplicates was measured at 550 nm.

In silico investigations

Molecular Dynamics Simulations. Desmond MD engine[2] was used for the MD simulation with OPLS4[3] force field. The system was solvated in an orthorhombic box (minimum distance of 10 Å to the edges from the protein). A temperature of 300 K was used for membrane patch pre-equilibration. The water was described with the TIP3P[4] model. The final systems comprised ~48 k atoms. All simulations were run in the NpT ensemble (T = 310 K, Nosé-Hoover method; p = 1.01325 bar, Martyna-Tobias-Klein method) with default Desmond settings. Reversible reference system propagator algorithms (RESPA) integrator with 2 fs, 2 fs, and 6 fs timesteps were used for bonded, near and far, respectively. The default value of 9 Å was used for the Coulombic cut-off. The system was relaxed using the default Desmond protocol before the production simulation. For lenvatinib, infigratinib and pemigatinib 8 systems were individually prepared and simulated: WT, N549K, N549D, E565A, E565G, V562L, V564F, and V564I.The simulation time of an individual complex (FGFR2–selected mutant–selected ligand) was 2 μs, resulting in 48 μs of the total simulation time (3 ligands x 8 FGFR2 systems x 2 μs). All production simulations were conducted with the same settings as mentioned above. The described in silico pipeline of data generation for analyses of FGFR2 mutation impact is visualised on Fig. 5B. The simulation interaction diagram of the Maestro package (Schrödinger, LLC, New York, NY) was used as basis for the simulation analysis.

Interaction analyses. The calculations of interaction patterns of lenvatinib, infigratinib and pemigatinib (Fig. 5 and Suppl. Table 2), as well as interaction networks analysis of Molecular brake (Suppl. Table 1), hydrophobic interaction frequency (Suppl. Table 3), and torsional conformations of rotatable bonds in the gate area and the back cleft (Suppl. Figures 13-15) were conducted with simulation interaction analysis tool of Maestro.

MM-GBSA energy calculations. The molecular mechanics energies with generalized Born and surface area continuum solvation (MM-GBSA) were calculated with Prime[5,6] thermal MM/GBSA (Figure 5, Suppl. Figures 7-12). Each 5th frame of MD was used for MM–GBSA calculations (402 complexes proceeded for an individual complex x 3 ligands x 8 FGFR2 systems). MM-GBSA calculations report energies for the ligand, receptor, and complex structures as well as energy

differences relating to strain and binding and are broken down into contributions from various terms in the energy expression. MM-GBSA ΔG Binding Energy = Complex − Receptor – Ligand.

Data visualization. Results were plotted with Seaborn library for Python 3.7 (https://doi.org/10.5281/zenodo.3629445). Protein structures were visualized with PyMOL (The PyMOL Molecular Graphics System, Version 2.0 Schrödinger, LLC.) Graphical representations of figures were arranged using Adobe Illustrator©.

Supplementary Results and Discussion

In silico interaction analysis of the FGFR-specific TKIs infigratinib and pemigatinib in comparison with the multikinase inhibitor lenvatinib

For these analyses, we initially selected the three different FGFR2 kinase mutations E565A, V564F, and N549K, which have repeatedly been detected in patients' tumors during treatment with infigratinib or pemigatinib,[7,8,9] and compared these drugs to lenvatinib. Expanding this scope, we incorporated four additional mutants (N549D, V562L, V564I, E565G) highlighted in recent work of Goyal et. al.[10], which have also been described in earlier studies[11-14], namely N549D, V562L, V564I, and E565G,. Our study covers an in-depth FGFR2 ligand interaction analysis, binding free energy and its components justified by MM-GBSA and ligand torsional profiles.

Increased interaction engagement of lenvatinib. Our results show a prevalent interaction engagement of lenvatinib compared to infigratinib or pemigatinib for both wild type and the kinase-mutated FGFR2 forms (Figure 5C, Suppl. Table 2). Lenvatinib phenoxy moiety interacts with the catalytic lysine (K517, 20% of the analyzed trajectory), while the carboxamide residue binds E534 in the αC-helix. Therefore, lenvatinib in WT FGFR2 targets all three main kinase (sub)pockets: front (A567 ~95%, E574 ~35%), back (E534 ~20%), and the gate area (D644 ~95%). Across FGFR2 mutant simulations, lenvatinib consistently demonstrates an advantageous interaction pattern, suggesting a minor influence on its prevalent activity. In the E565A mutant, e.g., lenvatinib shows three additional interactions compared to infigratinib and pemigatinib: hydrogen bond to E534 (αC helix, 30% of simulation time) and two water bridges: to linker residue N571 (28%) and E574 in αD helix (32%) (Figure 5C, Suppl. Table 2A). While the interaction advantage of lenvatinib in the V564F mutant is marginal, it forms a water bridge to a hinge residue E565 in over 50% of the simulation time, a unique feature absent in other systems.

An important structural feature of the kinase domain includes Asp-Phe-Gly, the so-called DFG-motif[15]. The DFG-motive classically exists in one of two states: the DFG-in state, a catalytically active conformation where the Asp residue facilitates ATP binding, and the DFG-out state, where the DFG motif extends away from the active side[16]. In the N549K mutant, lenvatinib exclusively forms a hydrogen bond with F645 in the DFG motif for 40% of the simulation time, alongside a bond with D644 of the DFG motif observed over 90% of the simulation time. In contrast, Infigratinib and pemigatinib exhibit D644 bonds with <80% frequency (Figure 5C, Suppl. Table 2A).

Furthermore, lenvatinib consistently demonstrates predominant interaction engagement in the N549D, V562L, V564I, and E565G mutants as well (Suppl. Table 2B). This is coupled with its notably enhanced hydrophobic interaction affinity (Suppl. Table 3B). Importantly, among these mutants, lenvatinib-bound systems uniquely exhibit a salt bridge interaction with the αC-helix residue E534 (Suppl. Table 2B). Additionally, water-mediated interactions are also specific to

lenvatinib, further accentuating its superior adaptability to various FGFR2 mutations (Suppl. Table 2B).

MM-GBSA energy proposes two potential binding conformations of lenvatinib in the V564F FGFR2 mutant. Our results revealed two potential binding conformations, according to their binding energies, for lenvatinib–V564F (Supplementary Fig. 7, displaying the bimodal distribution in turquoise). The higher energy binding mode (~ -2.7 ((kcal/mol)/HAC) is beside the median value of infigratinib–V564F binding energy (-2.9 ((kcal/mol)/HAC). Nevertheless, the lower energy minimum (~ -3.8 ((kcal/mol)/HAC) suggests that lenvatinib-V564F can switch the interaction pattern by forming an abundant number of short-term interactions that are not shown by the interaction analysis (under 20% of simulation time). The hydrogen bonding correction component indicates the same trend with high variation in values: (from -0.5 kcal/mol to -3.5 kcal/mol). Therefore, it can be anticipated that with more extended MD simulations, lenvatinib could embrace a diverse conformation, suggesting a solid but diverse interaction pattern, resulting in superior inhibitory performance compared to infigratinib. It is important to notice that lenvatinib-bound systems maintain consistently lower energy values than infigratinib-bound systems, reflecting the overall tighter binding of lenvatinib (Supplementary Fig. 7).

Further, the ligand strain energy values, which indicate the energy penalty to adopt the conformation of the ligand into the binding conformation, are lowest in lenvatinib-bound systems (Supplementary Fig. 9). In other words, the lower the value, the easier the ligand transits to the binding conformation. In other components, forming Gibbs free energy of binding the values remain in a close range for lenvatinib, infigratinib and pemigatinib in Van der Waals energy component contribution (Supplementary Fig. 10) and π–π packing (Supplementary Fig. 12), emphasizing diverse values in V564F mutant systems. Moreover, the median values of the hydrogen-bonding energy component are in agreement with the investigated systems, with lenvatinib showing the most significant deviation in values, protruding towards -3.5 kcal/mol (Supplementary Fig. 11). This observation also suggests that lenvatinib is engaged in a larger number of short-term interactions that contribute to its overall better inhibitory performance.

FGFR2 Elements of the FGFR2 molecular brake motif display a conserved behavior independently from mutations. The E565 and E549 residues compose an autoinhibitory substructure, called 'molecular brake' – an established dynamic element of FGFRs located in the kinase hinge region[17]. Mutations in this region were shown to disrupt the autoinhibitory network of hydrogen bonds in the molecular brake region, leading to profound kinase activation[17]. Due to its relevance for FGFR2[18] we investigated the stability of the hydrogen bond network of the molecular brake by MD simulations. Interestingly, for all ligand-mutant/WT combinations investigated, the interaction pattern remained consistent and showed no considerable difference (Supplementary Tab. 1). Moreover, in all systems upon the E565A mutation, a N549–A565

hydrogen bond was noted in more than 80% of the simulation time. Therefore, we suggest that the molecular brake stabilization is not crucial for the inhibitory activity in the mutants.

Interaction with the hydrophobic regulatory spine (R-spine). Another well-established dynamic element among protein kinases is the hydrophobic regulatory R-spine, which are formed by the four conserved residues (RS1–RS4) in the catalytically active form of a protein kinase[19,20]. While 55% of the FDA-approved small-molecule kinase inhibitors display ligand–RS3 hydrophobic interactions, mutations in R-spine lead to the loss of these contacts and are associated with treatment resistance[21]. To investigate a potential ligand activity–mutation correlation, we conducted a quantitative analysis of the hydrophobic contacts, which are formed by the ligands with the R-spine (Supplementary Table 3). Lenvatinib showed the highest number of overall hydrophobic interactions in comparison to both infigratinib and pemigatinib, which was consistently observed in all investigated systems. While the R-spine engagement was comparable between lenvatinib- and Infigratinib-bound systems, RS4 engagement was dominant in lenvatinib–bound systems. Lenvatinib-bound systems encompassed an average of 200 hydrophobic interactions against a maximum of 3 in infigratinib systems. Nevertheless, infigratinib displayed increased contacts to RS1–RS4 compared to lenvatinib in the E565A FGFR2 mutant.

Supplementary Figures

Supplementary Figure 1

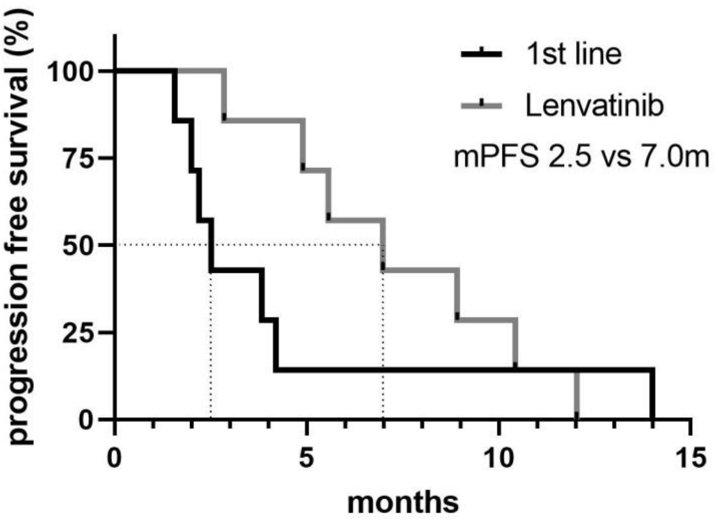

PFS comparison of first line therapies to the later line treatment with lenvatinib. Kaplan-Meier-survival curves demonstrating PFS during the treatment with lenvatinib compared to the PFS during the prior first-line therapy in the patient cohort (n=7).

Supplementary Figure 2

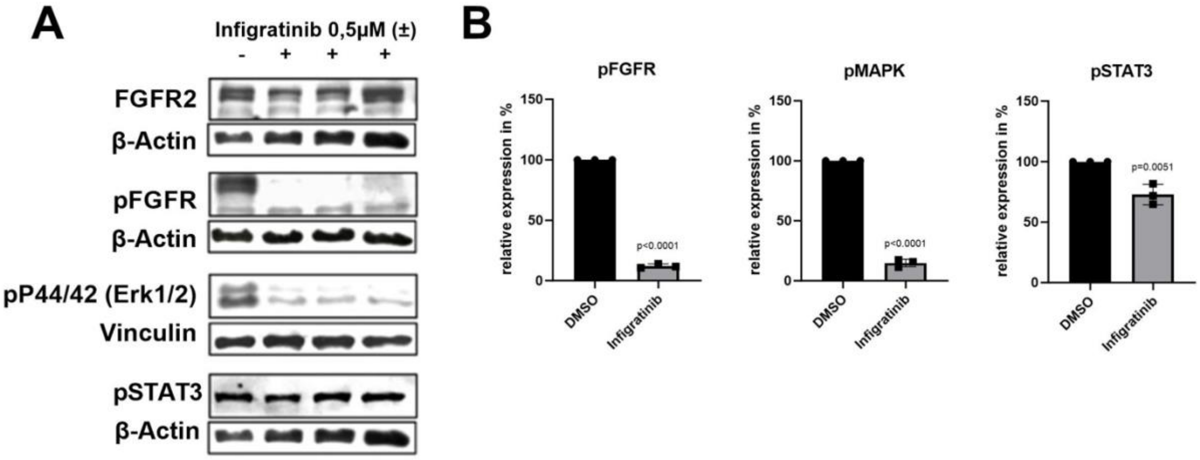

Western blot analysis of the FGFR2 pathway in FGFR2-AHCYL2 transfected NIH/3T3 cells after 24h treatment with 0.5µM infigratinib **(A)**. Densitometric analyses **(B)** of pFGFR, pMAPK and pSTAT3 in FGFR2-AHCYL2-NIH/3T3 cell line after treatment with infigratinib, graphs show mean ± SD, p values were calculated using two-sided Student´s t-test. Densitometric signal from DMSO bands were normalized to 100% in each repetition (n=3 biological replicates).

Supplementary Figure 3

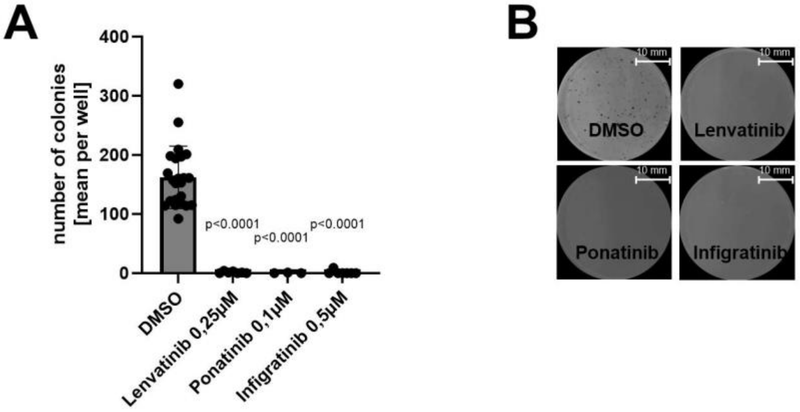

Tyrosine kinase inhibitors with FGFR2-inhibiting capacity prevent anchorage-dependent growth of FGFR2-fusion expression cell lines. **(A)** Quantification of soft agar colony formation after 21 days of NIH3T3 expressing FGFR2-AHCYL2 fusion in the presence of Lenvatinib,

Infigratinib and Ponatitinib. **(B)** Representative images of soft agars after 21 days of treatment with DMSO or the indicated TKI. Bars represent mean ± SD. ****$P \leq 0.0001$ compared to DMSO treated FGFR2-AHCYL2-NIH3T3 cells using One-Way-ANOVA with Dunnett's multiple comparison test (n=3 vials with ponatinib, n=6 vials with lenvatinib and infigratinib, n=21 vials with DMSO examined over 3 independent experiments)

Supplementary Figure 4

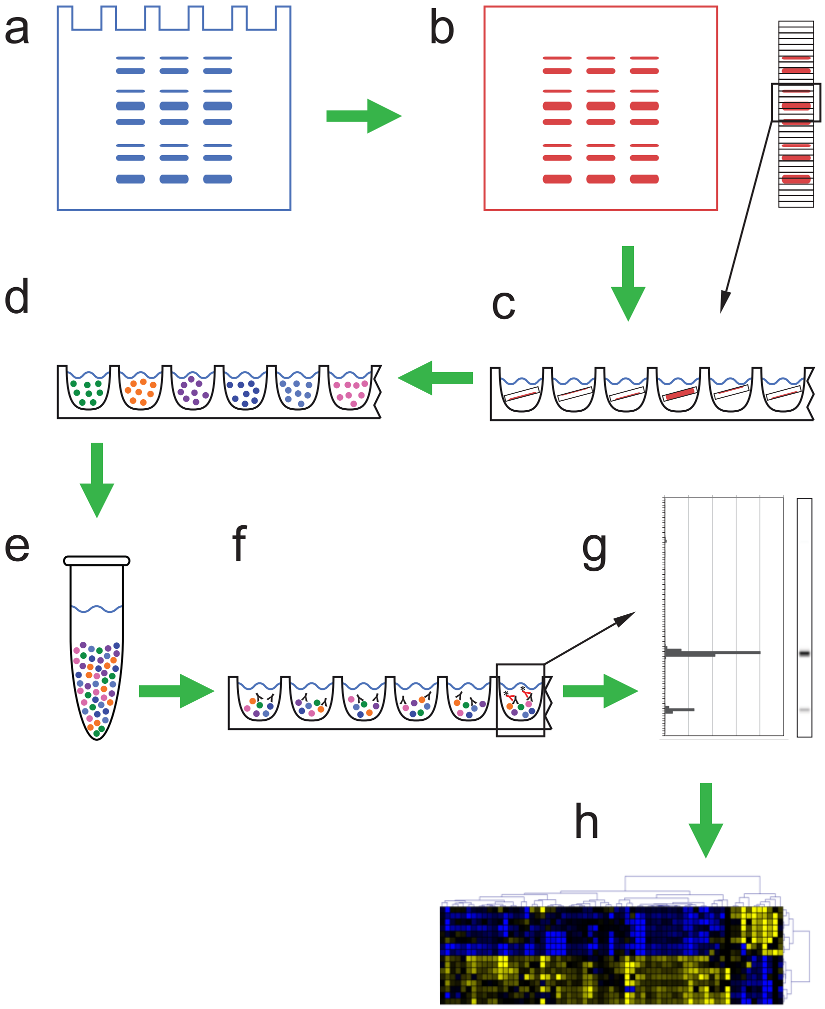

DigiWest workflow (modified (h was added) from Treindl F et al, Nature Communication 2016, as per the license terms https://creativecommons.org/licenses/by/4.0/)**: (A)** SDS-PAGE. **(B)** Western blot and biotinylation of proteins with subsequent cutting of each lane into 96 fractions. **(C)** Elution of molecular weight protein fractions into 96-well plates. **(D)** Protein loading onto magnetic color-coded neutravidin-coated Luminex® beads. **(E)** Pooling of beads. **(F)** Bead mixes yield hundreds of antibody-based immunoassays. **(G)** Readout on a Luminex® instrument and digital signal integration. **(H)** DigiWest protein profile.

A complete list of employed antibodies is given in the Supplementary Data 1.

Supplementary Figure 5

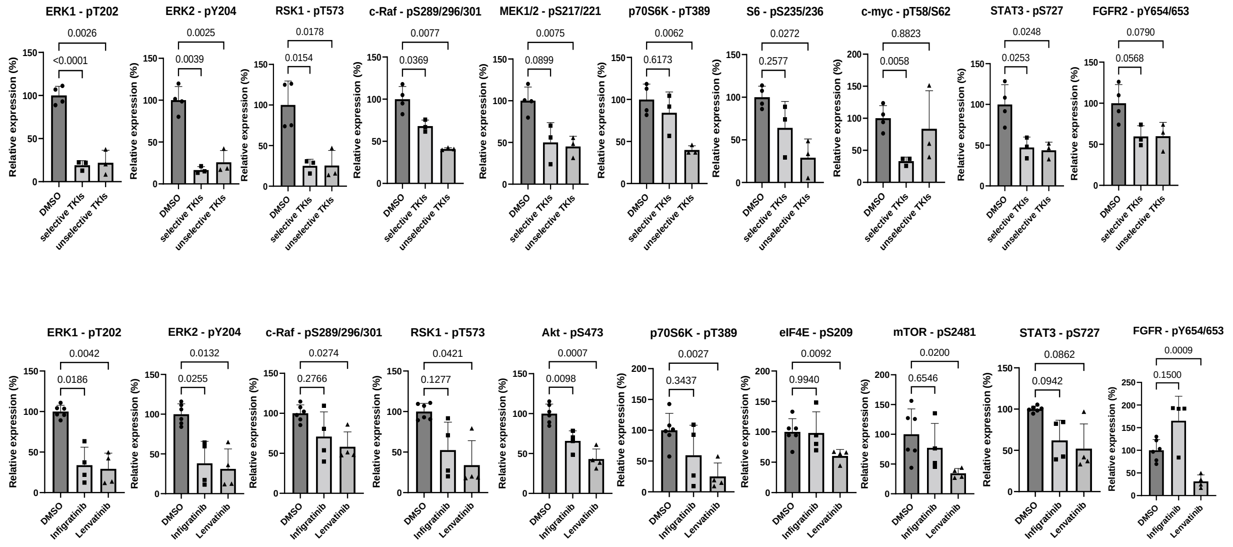

Effect of selective and unselective FGFR2 inhibiting TKIs on selected downstream targets on key downstream phosphorylation events: DigiWest analysis of selected downstream targets in (A) F-AHCYL2 WT and (B) F-AHCYL2_p.V564F cells. Data are shown relative to their respective control and each condition was compared to DMSO using Welch´s ANOVA with Dunnett´s Multiple Comparisons. Bars represent mean ± SD, n = 4 (DMSO) and n =3 (selective and unselective TKIs) biologically independent samples for F-AHCYL2 WT cells and n = 6 (DMSO) and n = 4 (infigratinib and lenvatinib) biologically independent samples for F-AHCYL2_p.V564F cells

Supplementary Figure 6

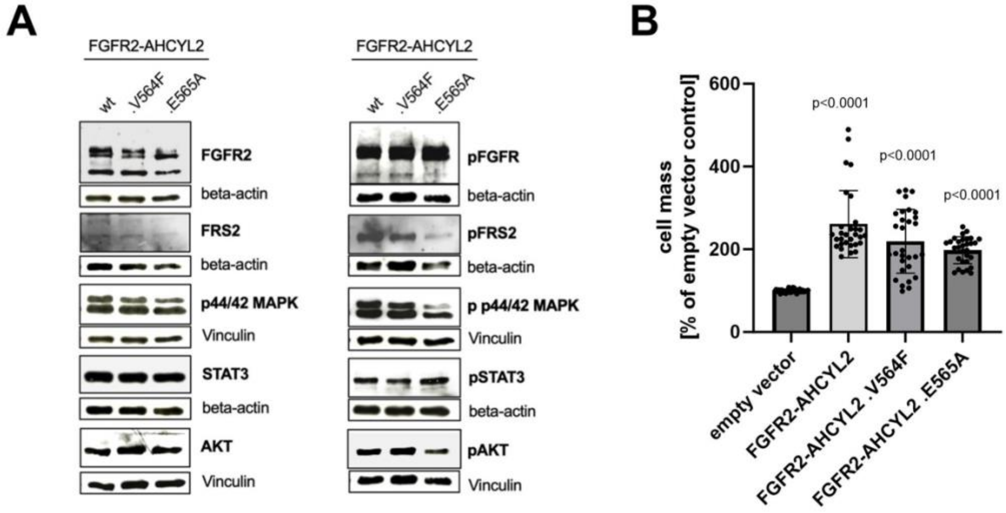

Comparison of F-AHCYL2_V564F, F-AHCLY2_E565A and F-AHCYL2_WT. (A) Western blot analysis of FGFR2 downstream signaling proteins in NIH3T3 cells expressing FGFR2-AHCYL2 as wild type or with additionally introduced point mutation in the kinase domain (Western Blot was repeated at least three times for each depicted signaling protein). **(B)** Proliferation analyses of FGFR2 fusion-expressing cell lines using SRB assays after 7 days in culture, graphs show mean ± SD, p values were calculated using One-Way-ANOVA with Dunnett's multiple comparison test Bars represent mean ± SD (n=30 vials examined over 10 independent experiments).

Supplementary Figure 7

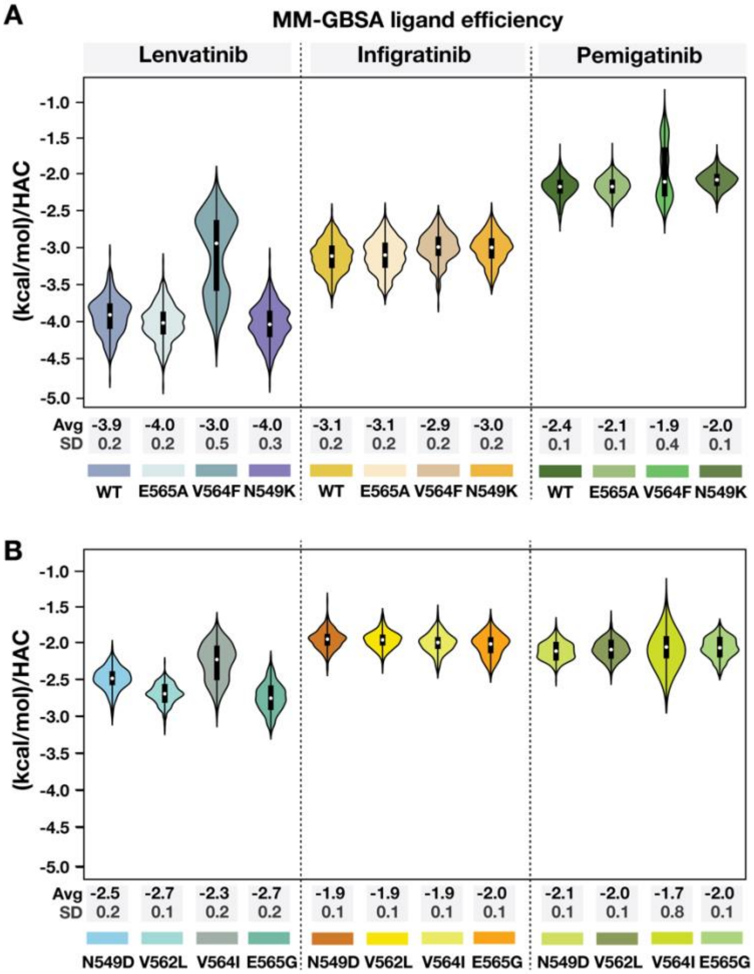

Lenvatinib-bound systems maintain superior ligand efficiency and display two binding conformations in V564F mutant.

Ligand efficiency, derived from MM-GBSA analysis of MD simulations is shown in violin plots. The timescale for each drug–mutant combination is 2us. The median is represented by a white circle, the interquartile range (IQR) by a central black bar, and data within 1.5 times the IQR by a thin black line. Kernel density estimations on either side of the black line illustrate data distribution shape, with wider sections indicating higher probability of population values. Heavy atom count (HAC) is used for ligand efficacy evaluation. HAC is a measure of the number of non-hydrogen atoms in the ligand molecule. It is employed as a normalization factor in MM-GBSA calculations to account for ligands with different molecular sizes. **(A)** Ligand efficacy comparison among drug-FGFR2 combinations: WT, E565A, V564F, and N549K mutants. **(B)** Extended ligand efficacy analysis in additional mutations N549D, V562L, V564I, and E565G.

Supplementary Figure 8

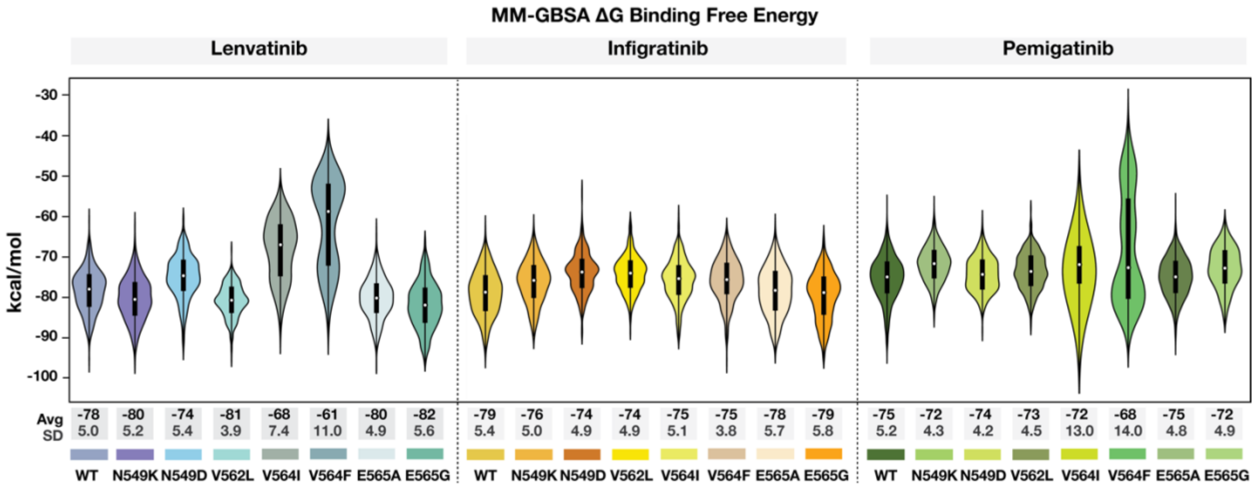

ΔG Binding Free Energy in drug-FGFR2 interactions estimates the strength of protein-ligand binding.

MM-GBSA derived ΔG Binding Free Energy is calculated from 2µs MD simulations of selected drug-FGFR2-mutant systems, illustrated with violin plots. The white circle represents the median. The thick black bar in the center represents the interquartile range (IQR), and the thin grey line represents the rest of the data within 1.5 times of the IQR. On each side of the grey line is a kernel density estimation to show the distribution shape of data. Wider sections represent a higher probability that members of the population will take on the given value. WT – wild type

Supplementary Figure 9

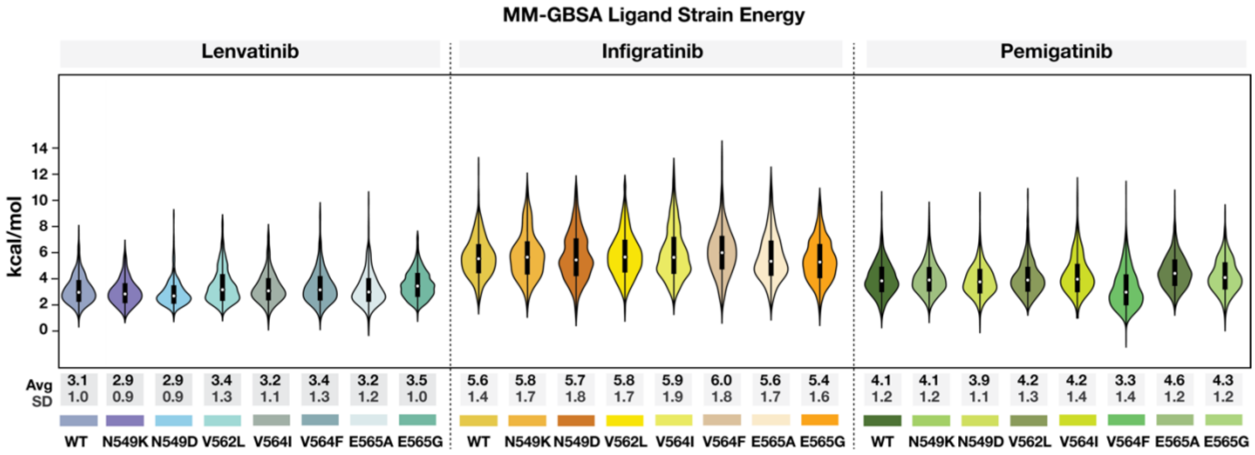

MM-GBSA ligand strain energy in drug-FGFR2 systems.

Ligand Strain Energy describes structural and energetic distortions that drug undergoes when binding to a protein's active site. It quantifies the energetic cost associated with adapting or rearranging the conformation to achieve optimal interactions within the binding pocket. Such adaptation includes conformational changes (rotations, bending, or stretching of the ligand's bonds), steric interactions or clashes, distortion of ligand electronic structure (adjustments in bond lengths, angles, and dihedrals), and hydrogen bonding and interaction matching.
MM-GBSA derived ligand strain energy is calculated from 2µs MD simulations of selected drug-FGFR2-mutant systems, illustrated with violin plots. The white circle represents the median. The thick black bar in the center represents the interquartile range (IQR), and the thin grey line represents the rest of the data within 1.5 times of the IQR. On each side of the grey line is a kernel density estimation to show the distribution shape of data. Wider sections represent a higher probability that members of the population will take on the given value. WT – wild type.

Supplementary Figure 10

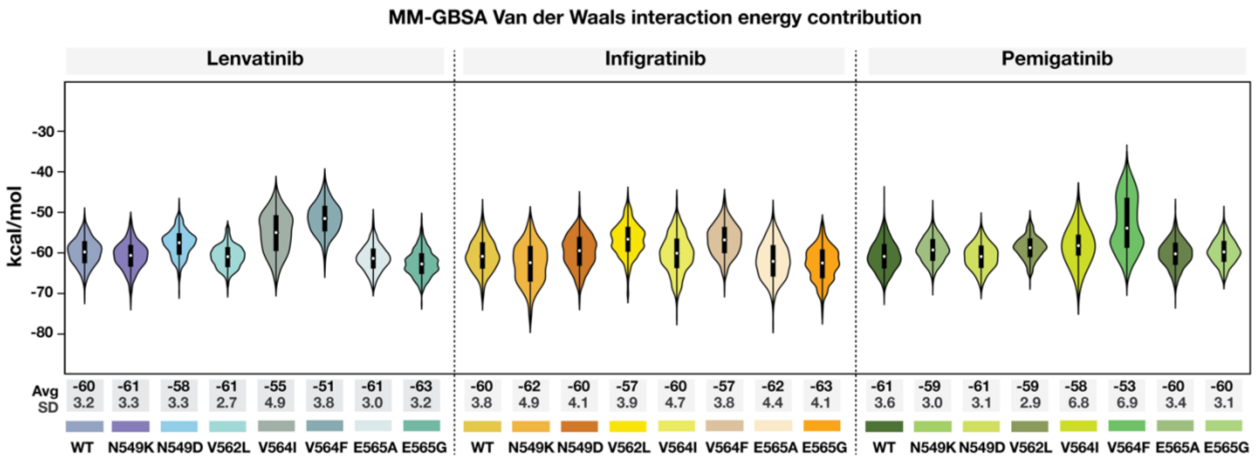

Van der Waals interaction energy in drug-FGFR2 systems.

Van der Waals energy contribution represents the energy associated with favourable interactions between nonpolar groups in the drug and protein, justifying attractive van der Waals forces and repulsive steric clashes.

MM-GBSA derived Van der Waals energy contribution is calculated from 2µs MD simulations of selected drug-FGFR2-mutant systems, illustrated with violin plots. The white circle represents the median. The thick black bar in the center represents the interquartile range (IQR), and the thin grey line represents the rest of the data within 1.5 times of the IQR. On each side of the grey line is a kernel density estimation to show the distribution shape of data. Wider sections represent a higher probability that members of the population will take on the given value. WT – wild type

Supplementary Figure 11

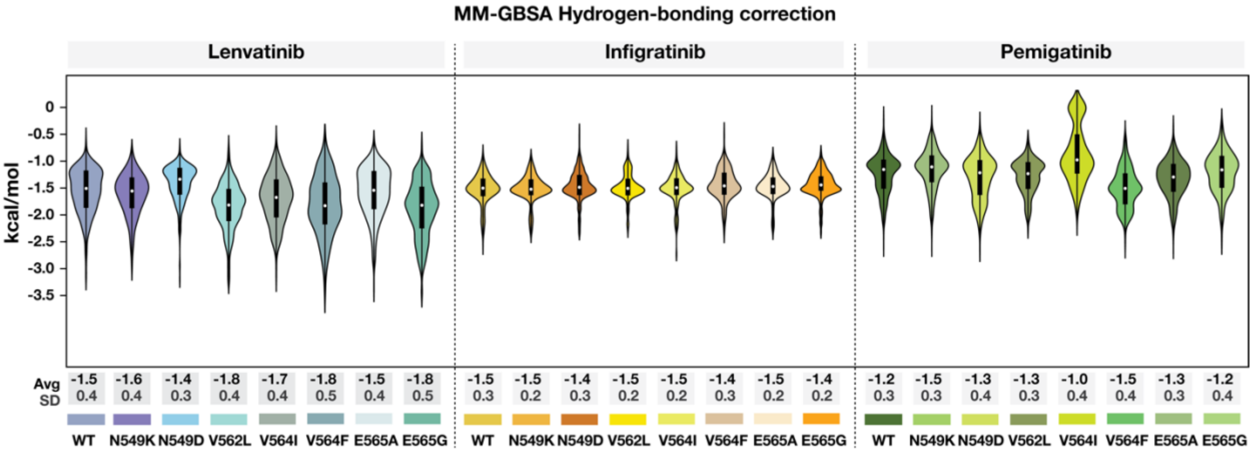

Hydrogen Bond interaction energy in drug-FGFR2 systems.

Hydrogen bond energy contribution justifies hydrogen bond contributions to binding dynamics. These interactions involve hydrogen atoms bridging electronegative atoms in ligand and protein, bolstering binding affinity.

MM-GBSA derived hydrogen bond energy contribution is calculated from 2µs MD simulations of selected drug-FGFR2-mutant systems, illustrated with violin plots. The white circle represents the median. The thick black bar in the center represents the interquartile range (IQR), and the thin grey line represents the rest of the data within 1.5 times of the IQR. On each side of the grey line is a kernel density estimation to show the distribution shape of data. Wider sections represent a higher probability that members of the population will take on the given value. WT – wild type

Supplementary Figure 12

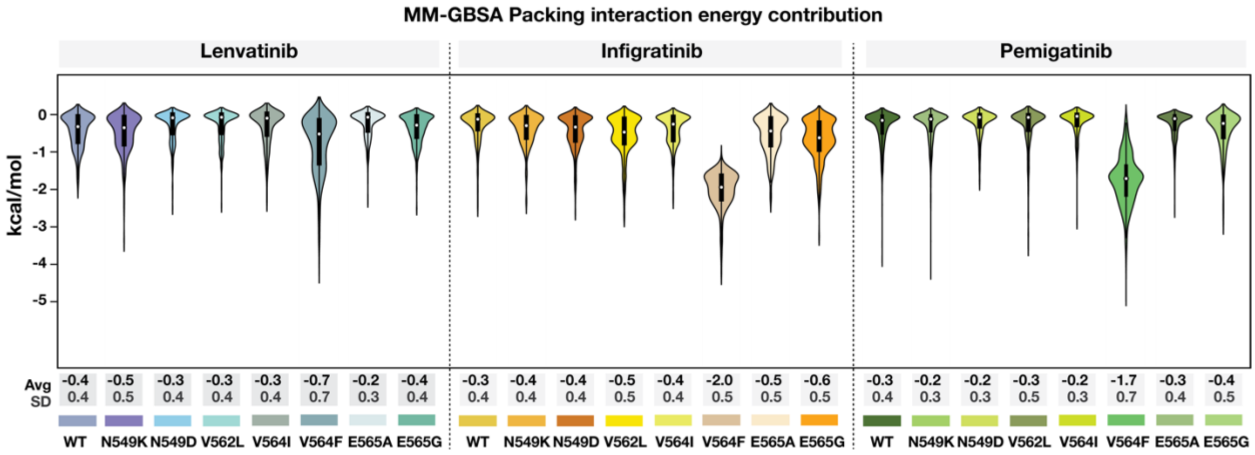

π–π packing energy in drug-FGFR2 systems.

π–π packing interaction energy considers the steric complementarity between the drug and FGFR2 binding site. It accounts for favourable interactions when hydrophobic or nonpolar groups in the ligand and protein come into close contact, creating a well-packed binding interface. MM-GBSA derived **π–π** energy contribution is calculated from 2µs MD simulations of selected drug-FGFR2-mutant systems, illustrated with violin plots. The white circle represents the median. The thick black bar in the center represents the interquartile range (IQR), and the thin grey line represents the rest of the data within 1.5 times of the IQR. On each side of the grey line is a kernel density estimation to show the distribution shape of data. Wider sections represent a higher probability that members of the population will take on the given value. WT – wild type

Supplementary Figure 13

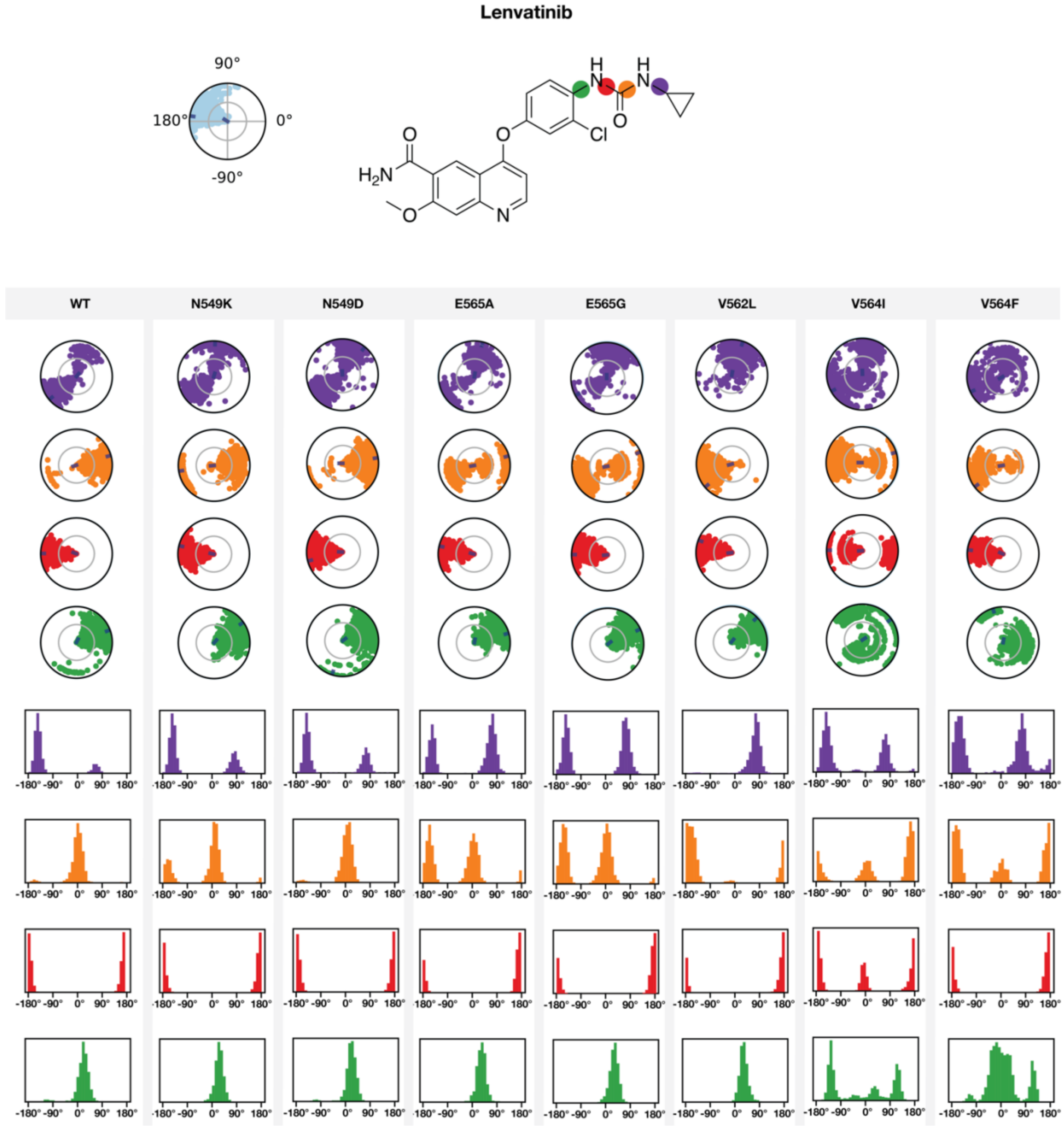

Lenvatinib torsional profile in the gate area and the back cleft. Polar plots illustrate the dynamic torsional conformations of Lenvatinib over time. The radial coordinate represents the MD simulation duration and angular coordinate indicates the torsional angle. Bar charts quantifying the torsional probabilities across different angles, reflecting their distribution throughout the simulation. Color coding corresponds to specific rotatable bonds in Lenvatinib structure. The studied mutations are highlighted with grey horizonal line

Supplementary Figure 14

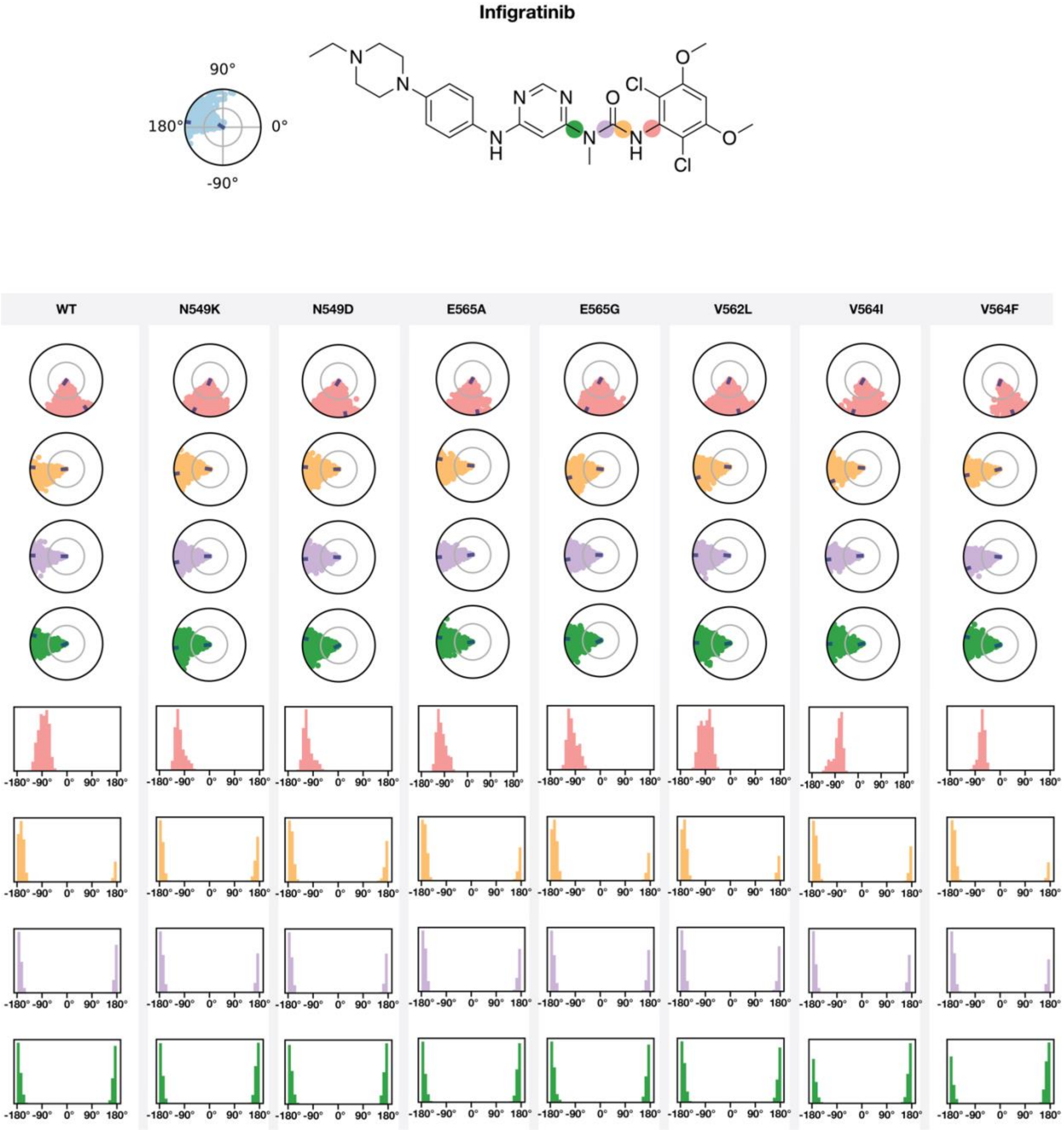

Infigratinib torsional profile in the gate area and the back cleft. Polar plots illustrate the dynamic torsional conformations of Infigratinib over time. The radial coordinate represents the MD simulation duration and angular coordinate indicates the torsional angle. Bar charts quantifying the torsional probabilities across different angles, reflecting their distribution throughout the simulation. Color coding corresponds to specific rotatable bonds in Infigratinib structure. The studied mutations are highlighted with grey horizonal line

Supplementary Figure 15

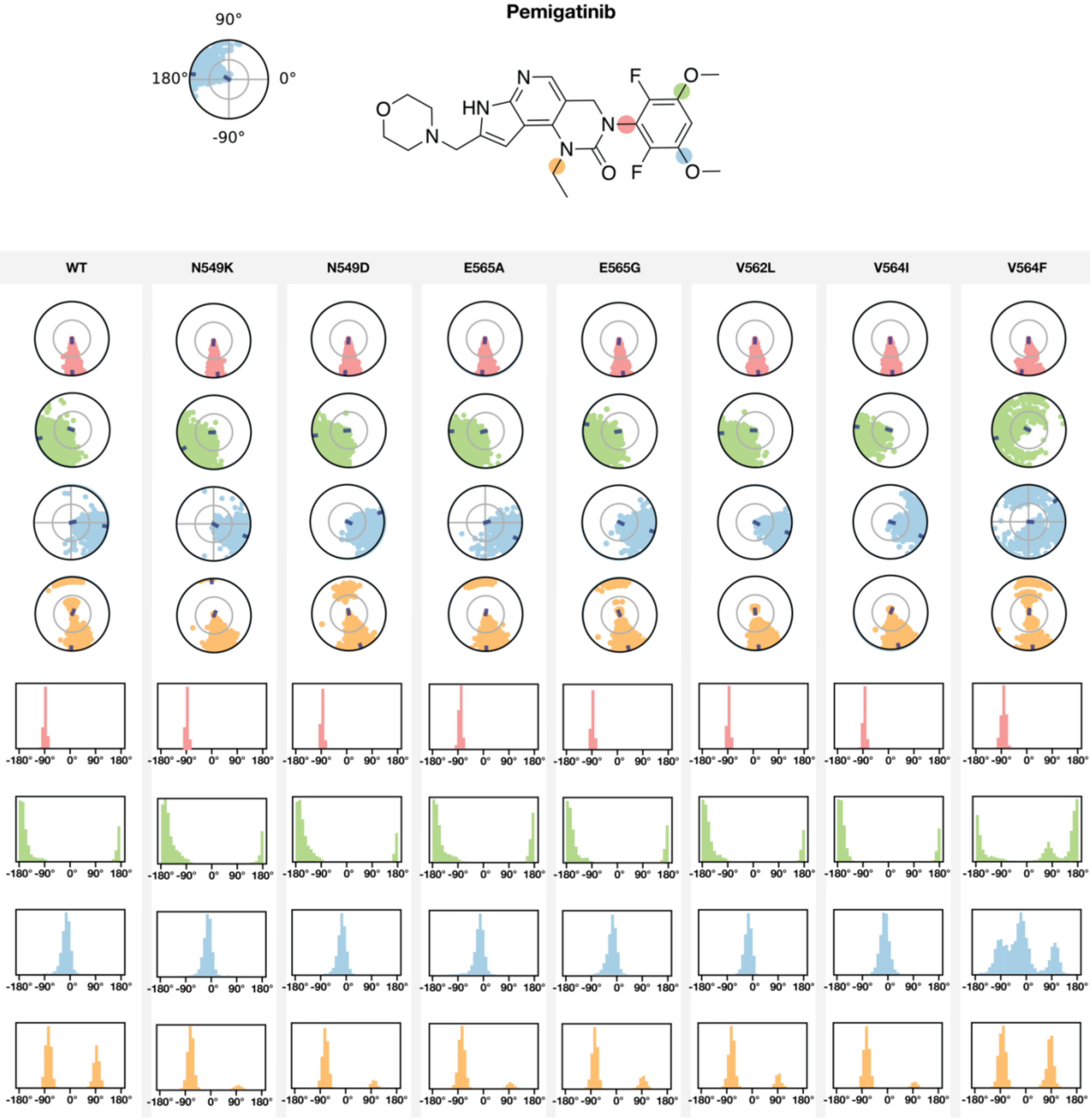

Pemigatinib torsional profile in the gate area and the back cleft. Polar plots illustrate the dynamic torsional conformations of Pemigatinib over time. The radial coordinate represents the MD simulation duration and angular coordinate indicates the torsional angle. Bar charts quantifying the torsional probabilities across different angles, reflecting their distribution throughout the simulation. Color coding corresponds to specific rotatable bonds in Pemigatinib structure. The studied mutations are highlighted with grey horizonal line.

Supplementary Figure 16

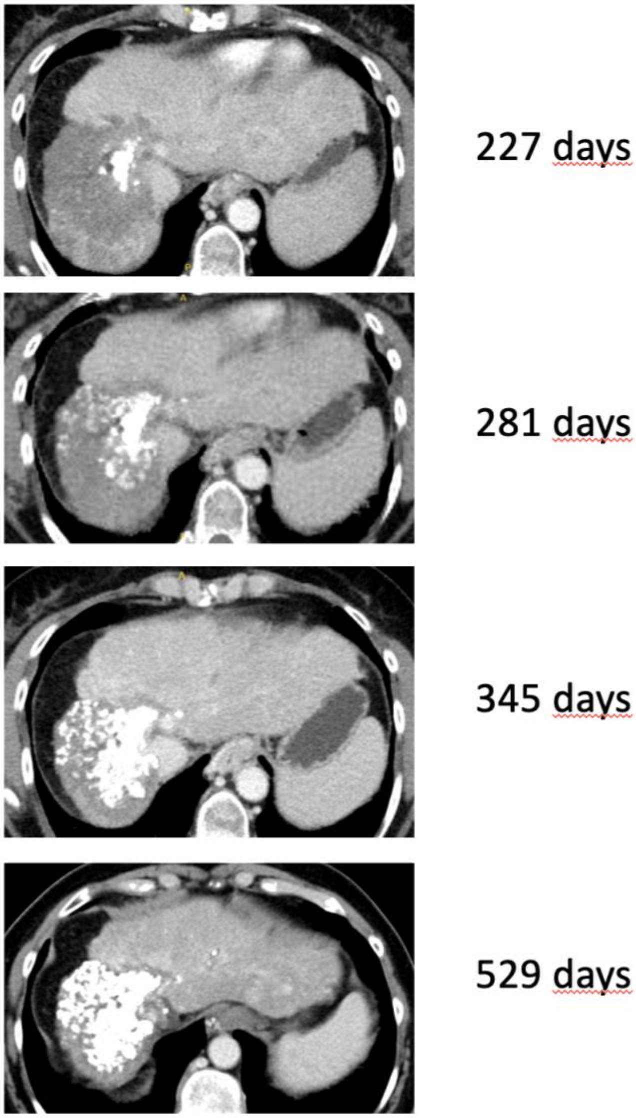

Hepatic calcification during treatment with pemigatinib. During the treatment with pemigatinib increased intrahepatic calcification was noted on repeated CT scannings. Hyperphosphatemia was successfully treated with a low-phosphate diet and phosphate binders, with most phosphate measurements during the therapy with pemigatinib being in the normal range. Nevertheless, the calcification continually increased and did not reverse after the treatment with pemigatinib was discontinued. Shown are the time period since start of pemigatinib in days.

Supplementary Tables

Supplementary Table 1

FGFR2 residue	Kinase subunit	WT			E565A			V564F			N549K		
		L	I	P	L	I	P	L	I	P	L	I	P
Molecular brake residue N549													
H544	β-sheet l	31% WB BB	90% HB BB	53% HB BB	48% WB BB	35% HB BB	55% HB BB	37% HB BB	95% HB BB	35% HB BB			
I547	β-sheet l	50% WB BB			50% WB BB	37% WB BB	39% WB BB						
E565	Brake	61% HB SC	93% HB SC	98% HB SC				61% HB SC	96% HB SC	94% HB SC	80% HB SC	90% HB SC	50% HB BB
K641	Brake												
A565	mutant				98% HB BB	98% HB BB	88% HB BB						
Molecular brake residue E565													
N549	Brake	94% HB BB	93% HB BB	98% HB BB	98% HB BB	98% HB BB	88% HB BB	90% HB BB	87% HB BB	86% HB BB			
K641	Brake	20% WB BB	48% HB SC	47% HB SC				43% HB SC	47% HB SC	40% HB SC	36% HB SC	45% HB SC	34% HB SC
K549	Mutant										94% HB BB	73% HB BB	69% HB BB
Molecular brake residue K641													
K545	b. l	53% HB BB	81% HB BB	60% HB BB	80% HB BB	81% HB BB	82% HB BB	32% HB BB	80% HB BB	36% HB BB			
E565	Triad	36% HB SC	96% HB SC	40% HB SC				86% HB SC	95% HB SC	90% HB SC	94% HB BB	90% HB SC	91% HB BB
L633	β-sheet VII	99% HB BB	86% HB BB	90% HB BB	99% HB BB	99% HB BB	99% HB BB	99% HB BB	99% HB BB	99% HB BB	98% HB BB	90% HB BB	97% HB BB

Lenvatinib, Infigratinib, and Pemigatinib, MD-derived interactions with FGFR2 molecular brake, show no significant changes upon mutations.

The presented percentages indicate the proportion of simulation time during which the ligand forms contacts with specific protein residues**.** The timescale for an individual drug–FGFR2-mutation system is 2μs. Interactions are shown when their contact strength exceeds 20% of the simulation time. L – Lenvatinib, I – Infigratinib, P – Pemigatinib; HB – hydrogen bond, WB – water bridge, SB – salt bridge, BB –backbone, SC – side chain. (i.e., HB SC – hydrogen bond from the ligand to side chain of a residue in the protein). b.l. = loop connecting αC-helix to β-sheet IV.

Supplementary Table 2

A

FGFR2r esidue	Kinase subunit	WT			N549K			V564F			E565A		
		L	I	P	L	I	P	L	I	P	L	I	P
L487	β-sheet I	23% WB BB			26% WB BB					22% WB SC			
K517	β-sheet III	20% Pi-cat	29% WB SC	45% WB SC		90% Pi-cat	52% HB SC			22% WB	22% Pi-cat	73% Pi-cat	23% Pi-cat
E534	αC helix	20% HB SC									31% HB SC		
E565	Hinge							55% WB					
Y566	Hinge									52% HB SC			
A567	Hinge	96% HB BB	99% HB BB	99% HB BB	26% HB BB	99% HB BB	86% HB BB	34% HB BB	99% HB BB	75% HB BB	94% HB BB	99% HB BB	83% HB BB
N571	Linker					22% WB BB					28% WB SC		
E574	αD helix	35% WB SC			25% WB SC		29% SB				32% WB SC		
D644	DFG	96% HB BB	76% HB BB	78% HB BB	93% HB BB	79% HB BB	60% HB BB	54% HB BB	77% HB BB	40% HB BB	99% HB	75% HB BB	70% HB
F645	DFG				41% HB BB				46% Pi-Pi				

FGFR2r esidue	Kinase subunit	N549D			V562L			V564I			E565G		
		L	I	P	L	I	P	L	I	P	L	I	P
L487	β-sheet I	23% WB BB									23% WB		
K517	β-sheet III	32% Pi-cat	79% Pi-cat	51% WB SC		21% Pi-cat	43% WB SC	31% Pi-cat*	44% WB SC	51% WB SC	21% Pi-cat	62% Pi-cat	55% WB SC
E534	αC helix				40% HB SC			30% HB SC			29% HB SC		
E565	Hinge												
Y566	Hinge												
A567	Hinge	96% HB BB	80% HB BB	92% HB BB	96% HB BB	77% HB BB	92% HB BB	93% HB BB	85% HB BB	86% HB BB	96% HB BB	85% HB BB	58% HB BB
N571	Linker				23% WB SC								
E574	αD helix			31% SB	21% WB SC		39% SB	27% WB SC			29% HB SC		
D644	DFG	95% HB BB	72% HB BB	55% HB BB	99% HB BB	82% HB BB	71% HB BB		77% HB BB	56% HB BB	99% HB BB	70% HB BB	93% HB BB
F645	DFG							21% WB BB			23% WB		

B

Lenvatinib, infigratinib and pemigatinib interaction patterns with FGFR2 WT and selected mutants derived from molecular dynamics simulations. (A) Interactions comparison among drug-FGFR2 combinations WT, E565A, V564F, and N549K mutants. **(B)** Interaction analysis in additional mutations N549D, V562L, V564I, and E565G. The percentages give the fraction of the simulation time that the ligand is in contact with protein residues. Molecular dynamics timescale

for individual replica is 2µs. Minimum contact strength displayed 20%. L – Lenvatinib, I– Infigratinib, P – Pemigatinib; HB – hydrogen bond, WB – water bridge, SB – salt bridge, BB – backbone, SC – side chain. (i.e., HB SC – hydrogen bond from the ligand to side chain of a residue in the protein). *Second Lenvatinib interaction with K517 is 63% HB SC

Supplementary Table 3

A

FGFR2 residue	Kinase subunit	WT			N549K			V564F			E565A		
		L	I	P	L	I	P	L	I	P	L	I	P
	Total amount	6257	4757	3542	6183	4955	3791	4871	3630	2720	6289	5391	4024
L487	β-I	523	779	431	593	674	364	468	626	753	633	822	426
V495	β-II	657	207	83	7816	62	125	564	93	267	549	173	75
A515	β-III	1003	370	590	1059	995	706	175	229	383	1320	713	721
L531	αC-helix	25	6	2	6			9	8		11	39	2
M538	RS3	76	467	390	193	129	319	211	282	118	225	129	286
I548	b.l	140	175	356	149		492	570	276	81	313	333	448
L550	β-IV, RS4	193	12	1	106		11	180	2	2	244	3	3
V562	β-V	883	246	227	739	643	305	29	25	3	590	499	345
V564	GK	641	1016	466	698	1375	527	(F) 1175	(F) 952	(F) 429	947	1028	805
A567	Hinge	2		2	6	1	2	14		1	11		7
Y566	Hinge	129	312	5	324	225	3	187	337	179	234	435	15
L633	β-VII	1009	619	462	1200	260	495	891	540	368	1025	513	478
A643	x DFG	218	3		340	17	23	167	1	86	148	3	5
F645	DFG, RS2	564	544	520	19	368	419	210	268	37	31	680	407
L550	β-IV	189						9					

B

FGFR2 residue	Kinase subunit	N549D			V562L			V564I			E565G		
		L	I	P	L	I	P	L	I	P	L	I	P
	Total amount	6031	4760	3733	6425	4522	3450	4467	2064	3752	6197	5239	3484
L487	β-I	556	631	387	609	497	380	631	302	396	512	722	68
V495	β-II	508	116	105	594	75	87	282	39	90	627	170	
M497	β-II		4			10		1	3		1	10	640
A515	β-III	1216	955	695	1185	323	488	493	147	328	1094	598	5
L531	αC-helix	49	10					61	3	3	14	45	
M535	αC-helix	59						252			23		275
M538	RS3	43	188	425	655	513	440	67	188	329			
I548	b.l	54	160	377	683	283	421	163	119	384	524	335	4
L550	β-IV, RS4	94		15	212			110	1		258	10	187
V562	β-V	1001	512	325	(L) 69	(L) 167	(L) 171	262	95	143	406	440	447
V564	GK	905	1209	603	762	1094	493	(I) 839	(I) 461	(I) 776	872	1048	6
Y566	Hinge	253	205	2	294	313	58	229	120	10	357	449	
L633	β-VII	1200	295	604	1066	684	559	841	314	454	1101	611	
A643	x DFG	120	35	19	84	2	7	121		1	207	1	522
F645	DFG, RS2	5	426	170	199	559	341	109	271	833	9	624	
M537	αC-helix										189	221	

Hydrophobic interaction frequencies derived from molecular dynamics simulations. (A) Interactions comparison among drug-FGFR2 combinations: WT, E565A, V564F, and N549K mutants. **(B)** Extended interaction analysis in clinically relevant mutations: N549D, V562L, V564I, and E565G. The total amount indicates the amount of ligand–FGFR2 hydrophobic contacts detected in all MD frames for a specific system (Amount of frames for each system is 2000, timescale 2μs). Interactions that are occurring >50 times in at least one system are shown. L –

Lenvatinib, I– Infigratinib, P – Pemigatinib; Brake – autoinhibitory substructure 'molecular brake'. I–VIII = β-sheets I–VIII; g.l. = G-rich loop, b.l. = loop connecting αC-helix to IV; GK = gatekeeper; linker = loop connecting the hinge to αD-helix; c.l. = catalytic loop; xDFG = DFG-motif plus one preceding amino acid residue.

Supplementary Table 4

	IC_{50} pemigatinib[22]	IC_{50} infigratinib[23]	IC_{50} lenvatinib[24]
FGFR1	0.4nM	0.9nM	61nM
FGFR2	0.5nM	1.4nM	27nM
FGFR3	1nM	1nM	52nM
FGFR4	30nM	60nM	43nM
Ratio FGFR2/FGFR1	1.3	1.6	0.4

Inhibitor profile of pemigatinib, infigratinib, and lenvatinib for FGFR1-4. Selected publications show different inhibitor profiles for the two FGFR-specific TKIs pemigatinib and infigratinib, and the non-selective TKI lenvatinib.

Supplementary Table 5

Primary and secondary antibodies for Western blotting

Antibody	Distributor	Cat. No.	Dilution
FGFR2 (Rabbit mAb)	Cell Signalling	#23328	1:1000
pFGFR (Mouse mAb)	Cell Signalling	#3476	1:1000
FRS2 (Mouse mAb)	Santa Cruz	sc-17841	1:100
pFRS2 (Rabbit mAb)	Cell Signalling	#3861	1:1000
MAPK (P44/42) (Rabbit mAb)	Cell Signalling	#4695	1:1000
pMAPK (pP44/42) (Rabbit mAb)	Cell Signalling	#9101	1:1000
STAT3 (Rabbit mAb)	Cell Signalling	#4904	1:2000
pSTAT3 (Rabbit mAb)	Cell Signalling	#9131	1:1000
AKT (pan) (Rabbit mAb)	Cell Signalling	#4685	1:1000

pAKT (1/2/3)(pan) (Mouse mAb)	Santa Cruz	sc-514032	1:500
Beta-actin (Mouse mAb)	Cell Signalling	#3700S	1:1000
Vinculin (Mouse mAb)	Sigma-Aldrich	V9131	1:5000
Goat anti-Mouse IgG (H+L) Secondary Antibody, HRP	Invitrogen	#31430	1:1000
Goat anti-Rabbit IgG (H+L) Secondary Antibody, HRP	Invitrogen	#31460	1:1000

Supplementary References

1. Treindl F, *et al.* A bead-based western for high-throughput cellular signal transduction analyses. *Nat Commun* **7**, 12852 (2016).

2. Bowers KJ, *et al.* Scalable Algorithms for Molecular Dynamics Simulations on Commodity Clusters. In: *SC '06: Proceedings of the 2006 ACM/IEEE Conference on Supercomputing*) (2006).

3. Lu C, *et al.* OPLS4: Improving Force Field Accuracy on Challenging Regimes of Chemical Space. *J Chem Theory Comput* **17**, 4291-4300 (2021).

4. Jorgensen WL, Chandrasekhar J, Madura JD, Impey RW, Klein ML. Comparison of simple potential functions for simulating liquid water. *The Journal of Chemical Physics* **79**, 926-935 (1983).

5. Jacobson MP, *et al.* A hierarchical approach to all-atom protein loop prediction. *Proteins: Structure, Function, and Bioinformatics* **55**, 351-367 (2004).

6. Jacobson MP, Friesner RA, Xiang Z, Honig B. On the Role of the Crystal Environment in Determining Protein Side-chain Conformations. *Journal of Molecular Biology* **320**, 597-608 (2002).

7. Silverman IM, *et al.* Clinicogenomic Analysis of FGFR2-Rearranged Cholangiocarcinoma Identifies Correlates of Response and Mechanisms of Resistance to Pemigatinib. *Cancer Discov* **11**, 326-339 (2021).

8. Goyal L, *et al.* Polyclonal Secondary FGFR2 Mutations Drive Acquired Resistance to FGFR Inhibition in Patients with FGFR2 Fusion-Positive Cholangiocarcinoma. *Cancer Discov* **7**, 252-263 (2017).

9. Krook MA, *et al.* Fibroblast growth factor receptors in cancer: genetic alterations, diagnostics, therapeutic targets and mechanisms of resistance. *Br J Cancer* **124**, 880-892 (2021).

10. Goyal L, *et al.* Futibatinib for FGFR2-Rearranged Intrahepatic Cholangiocarcinoma. *New England Journal of Medicine* **388**, 228-239 (2023).

11. Goyal L, *et al.* Polyclonal Secondary FGFR2 Mutations Drive Acquired Resistance to FGFR Inhibition in Patients with FGFR2 Fusion-Positive Cholangiocarcinoma. *Cancer Discov* **7**, 252-263 (2017).

12. Goyal L, *et al.* TAS-120 Overcomes Resistance to ATP-Competitive FGFR Inhibitors in Patients with FGFR2 Fusion-Positive Intrahepatic Cholangiocarcinoma. *Cancer Discov* **9**, 1064-1079 (2019).

13. Nakamura IT, *et al.* Comprehensive functional evaluation of variants of fibroblast growth factor receptor genes in cancer. *NPJ Precis Oncol* **5**, 66 (2021).

14. Chen H, *et al.* A molecular brake in the kinase hinge region regulates the activity of receptor tyrosine kinases. *Mol Cell* **27**, 717-730 (2007).

15. Farrell B, Breeze AL. Structure, activation and dysregulation of fibroblast growth factor receptor kinases: perspectives for clinical targeting. *Biochem Soc Trans* **46**, 1753-1770 (2018).

16. Roskoski R, Jr. Classification of small molecule protein kinase inhibitors based upon the structures of their drug-enzyme complexes. *Pharmacol Res* **103**, 26-48 (2016).

17. Chen H, *et al.* A molecular brake in the kinase hinge region regulates the activity of receptor tyrosine kinases. *Mol Cell* **27**, 717-730 (2007).

18. Chen H, *et al.* Elucidation of a four-site allosteric network in fibroblast growth factor receptor tyrosine kinases. *Elife* **6**, (2017).

19. Kornev AP, Haste NM, Taylor SS, Eyck LF. Surface comparison of active and inactive protein kinases identifies a conserved activation mechanism. *Proc Natl Acad Sci U S A* **103**, 17783-17788 (2006).

20. Robinson R. Confirming the importance of the R-spine: new insights into protein kinase regulation. *PLoS Biol* **11**, e1001681 (2013).

21. Shevchenko E, Pantsar T. Regulatory spine RS3 residue of protein kinases: a lipophilic bystander or a decisive element in the small-molecule kinase inhibitor binding? Biochemical Society Transactions 50, 633-648 (2022).

22. Liu, P. C. C. et al. INCB054828 (pemigatinib), a potent and selective inhibitor of fibroblast growth factor receptors 1, 2, and 3, displays activity against genetically defined tumor models. PLoS One 15, e0231877 (2020).

23. Guagnano, V. et al. FGFR genetic alterations predict for sensitivity to NVP-BGJ398, a selective pan-FGFR inhibitor. Cancer Discov 2, 1118-1133 (2012).

24. Tohyama, O. et al. Antitumor activity of lenvatinib (e7080): an angiogenesis inhibitor that targets multiple receptor tyrosine kinases in preclinical human thyroid cancer models. J Thyroid Res 2014, 638747 (2014).

Uncropped Western Blot of supplementary Figures

Supplementary Figure 2

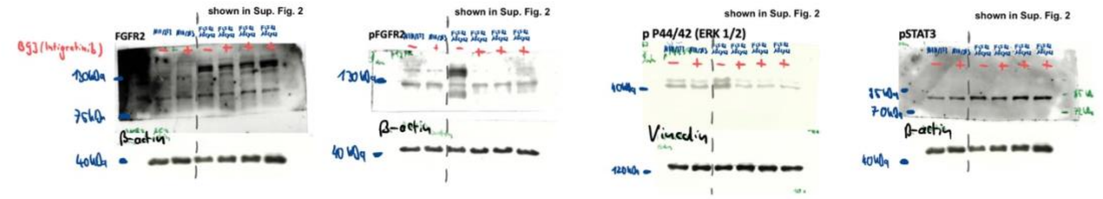

Supplementary Figure 6

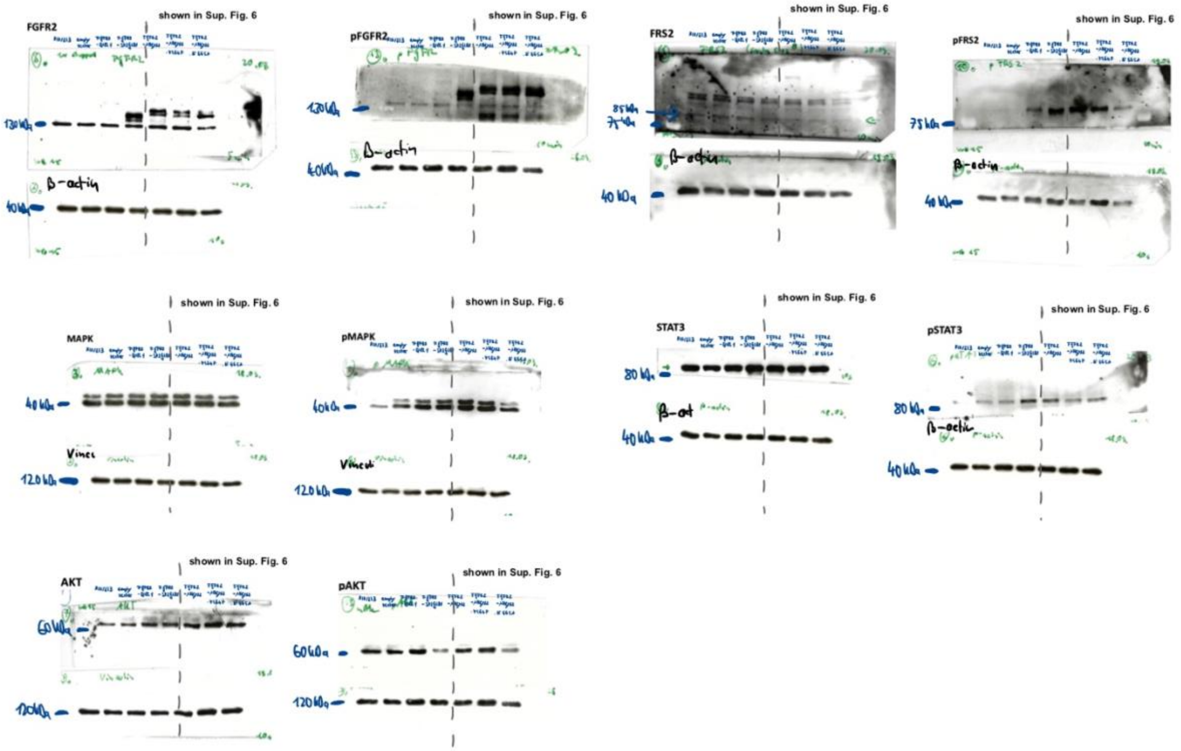

Supplement: Supplementary file 1 — Supplementary Information [file 41467_2024_45247_MOESM1_ESM.pdf]
