# Supplementary material for: The molecular interaction pattern of lenvatinib enables inhibition of wild-type or kinase-mutated FGFR2-driven cholangiocarcinoma
Source: Nat Commun. 2024 Feb 12;15:1287. doi: 10.1038/s41467-024-45247-6 (PMC10861557; doi:10.1038/s41467-024-45247-6)
Supplement: Supplementary file 3 — Description of Additional Supplementary Files [file 41467_2024_45247_MOESM3_ESM.pdf]

**Description of Additional Supplementary Files**

File Name: Supplementary Data 1
Description: *Additional data for DigiWest experiments*

1. Figure 3D/E: DigiWest protein profiling of NIH3T3 cells with FGFR2-AHCYL2 fusion.
2. Figure 4D/E: DigiWest protein profiling of NIH3T3 cells with FGFR2-AHCYL2 fusion and gate-keeper mutation p.V564F. Treatment with infigratinib or lenvatinib.
3. Figure 4F: DigiWest protein profiling of NIH3T3 cells with FGFR2-AHCYL2_p.V564F fusion - direct comparison of Lenvatinib vs Infigratinib
4. Supplementary Figure 5A: DigiWest: Relative expression of key downstream signaling phosphorylation events vs respective (experiment-matched) DMSO Control for F-AHCYL2 cells
5. Supplementary Figure 5B: DigiWest: Relative expression of key downstream signaling phosphorylation events vs respective (experiment-matched) DMSO Control for F-AHCYL2 p.V564F cells
6. DigiWest Antibody List
