# Supplementary material for: The molecular interaction pattern of lenvatinib enables inhibition of wild-type or kinase-mutated FGFR2-driven cholangiocarcinoma
Source: Nat Commun. 2024 Feb 12;15:1287. doi: 10.1038/s41467-024-45247-6 (PMC10861557; doi:10.1038/s41467-024-45247-6)
Supplement: Supplementary file 5 — Reporting Summary [file 41467_2024_45247_MOESM5_ESM.pdf]

nature portfolio

Corresponding author(s): Stephan Spahn and Michael Bitzer

Last updated by author(s): Nov 21, 2023

Reporting Summary

Nature Portfolio wishes to improve the reproducibility of the work that we publish. This form provides structure for consistency and transparency in reporting. For further information on Nature Portfolio policies, see our Editorial Policies and the Editorial Policy Checklist.

Statistics

For all statistical analyses, confirm that the following items are present in the figure legend, table legend, main text, or Methods section.

n/a	Confirmed	
☐	☒	The exact sample size (*n*) for each experimental group/condition, given as a discrete number and unit of measurement
☐	☒	A statement on whether measurements were taken from distinct samples or whether the same sample was measured repeatedly
☐	☒	The statistical test(s) used AND whether they are one- or two-sided<br>*Only common tests should be described solely by name; describe more complex techniques in the Methods section.*
☒	☐	A description of all covariates tested
☐	☒	A description of any assumptions or corrections, such as tests of normality and adjustment for multiple comparisons
☐	☒	A full description of the statistical parameters including central tendency (e.g. means) or other basic estimates (e.g. regression coefficient) AND variation (e.g. standard deviation) or associated estimates of uncertainty (e.g. confidence intervals)
☐	☒	For null hypothesis testing, the test statistic (e.g. F, t, r) with confidence intervals, effect sizes, degrees of freedom and P value noted<br>*Give P values as exact values whenever suitable.*
☒	☐	For Bayesian analysis, information on the choice of priors and Markov chain Monte Carlo settings
☐	☒	For hierarchical and complex designs, identification of the appropriate level for tests and full reporting of outcomes
☐	☒	Estimates of effect sizes (e.g. Cohen's *d*, Pearson's *r*), indicating how they were calculated

Our web collection on statistics for biologists contains articles on many of the points above.

Software and code

Policy information about availability of computer code

Data collection

DigiWest readout was perfomed on Luminex FlexMAP 3D using xPONENT v. 4.3.
in silico modeling:
Protein structure preparation – Protein preparation Wizard; part of Maestro Suite, version 2021.3 (Schrödinger LLC, NY)
Ligands preparation – LigPrep, part of Maestro Suite, version 2021.3
Ligand Docking – Glide, part of Maestro Suite, version 2021.3

Data analysis

GraphPad Prism 7 and 9
ImageJ (v.1.54k)
MEV 4.9.0
in silico modeling:
Molecular Dynamics Simulations - Desmond MD engine, OPLS4 force field; part of Maestro Suite, versions 2021.3 and 2023.1
Interaction analysis – simulation interaction analysis tool, Maestro Suite, version 2023.1
Torsional profiles – simulation interaction analysis tool, Maestro Suite, version 2023.1
MM/GBSA energy predictions – Prime thermal MM/GBSA, part of Maestro Suite, version 2023.1
Statistical evaluation of MM/GBSA energy – Seaborn library for Python, version 3.8.17
Structure visualisation – PyMOL Molecular Graphics System, Version 2.5.2 Schrödinger, LLC.

For manuscripts utilizing custom algorithms or software that are central to the research but not yet described in published literature, software must be made available to editors and reviewers. We strongly encourage code deposition in a community repository (e.g. GitHub). See the Nature Portfolio guidelines for submitting code & software for further information.

Data

Policy information about availability of data

All manuscripts must include a data availability statement. This statement should provide the following information, where applicable:

- Accession codes, unique identifiers, or web links for publicly available datasets
- A description of any restrictions on data availability
- For clinical datasets or third party data, please ensure that the statement adheres to our policy

All data generated or analyzed during this study are included in the published artice and its supplementary file. The datasets generated during the in-silico study are available in the Zenodo repository (DOI: 10.5281/zenodo.7456830). The available data includes the raw molecular dynamic trajectories and full-component MM-GBSA tables.

Research involving human participants, their data, or biological material

Policy information about studies with human participants or human data. See also policy information about sex, gender (identity/presentation), and sexual orientation and race, ethnicity and racism.

Reporting on sex and gender	Both self-reported males and females were included in this study. Of the eight patients, five were female. This ratio is expected, as several publication demonstrated that FGFR2 mutations are likely in female CCA patients.
Reporting on race, ethnicity, or other socially relevant groupings	We did not collect information about race, ethnicity or socially relevant groupings for this study.
Population characteristics	Eight individual cases of FGFR2-driven CCA are reported. Five were female, three male, age 34-73 (mean 55.5) years. The CCA of five patients harbored FGFR2-fusion proteins. Two had activating point mutations outside the kinase domain of FGFR2(p.C382R and p.S372C), and one tumor harbored an extracellular domain in-frame deletion (370_371delinsCys & DEL) of FGFR2.
Recruitment	All patients were presented to the Molecular Tumor Board (MTB) of Tubingen University. The MTB identified FGFR2 as a relevant target, however no approved drug or a clinical study were available at this point in time, therefore the MTB recommended lenvatinib, due to its availability and ability to inhibit FGFR2. Off-label treatments are usually part of recommendations by the MTB, which consists of an interdisciplinary team including experts in clinical and translational oncology, pathology, bioinformatics, molecular biology, radiology, and human genetics. All patients gave written informed consent to treatment with lenvatinib.
Ethics oversight	The translational study was reviewed and approved by the local ethics committee of the medical faculty of University Tubingen (714/2019BO2) and was conducted in accordance with the Declaration of Helsinki. Before genetic tumor analysis, patients were consulted by a specialist in clinical genetics. All patients gave written informed consent. After the MTB recommended lenvatinib, all included patients gave written informed consent to the treatment with lenvatinib.

Note that full information on the approval of the study protocol must also be provided in the manuscript.

Field-specific reporting

Please select the one below that is the best fit for your research. If you are not sure, read the appropriate sections before making your selection.

☒ Life sciences ☐ Behavioural & social sciences ☐ Ecological, evolutionary & environmental sciences

For a reference copy of the document with all sections, see nature.com/documents/nr-reporting-summary-flat.pdf

Life sciences study design

All studies must disclose on these points even when the disclosure is negative.

Sample size	This was a retrospective analyses of all patients with FGFR2-driven CCA treated with the TKI lenvatinib at the University-hospital Tubingen prior to the approval of pemigatinib for this indication. As outlined in the manuscript, prior to the approval of pemigatinib by the European Commission in 03/21, the MTB recommended lenvatinib in FGFR2-driven cholangiocarcinoma, because at this point in time no approved FGFR-inhibiting drug was available. Taken together, we treated eight patients with FGFR2-driven CCA with Lenvatinib. All were included in the presented study. For the sample size of experiments, we depicted the sample size separately for every experiment in the text or legend of figures and sub-figures.
Data exclusions	No patient was excluded.
Replication	Both technical and biological replications were done. The number of technical and biological repeats are depicted in the text and in the legend for each figure and sup. figure separately.

Randomization	This is a retrospective observational study in a patient group with FGFR2-driven cholangiocarcinoma. The MTB recommended lenvatinib, an alternative drug was not available for these patients.
Blinding	Prior to the approval of pemigatinib by the European Commission in 03/21, all patients with FGFR2-driven cholangiocarcinoma received the MTB recommendation for the treatment with lenvatinib. Due to the retrospective nature of our observational study and analysis, blinding of the investigators was not possible. Radiologic responses were assesed by an experienced board certificated radiologist according to RECIST 1.1. critera.

Reporting for specific materials, systems and methods

We require information from authors about some types of materials, experimental systems and methods used in many studies. Here, indicate whether each material, system or method listed is relevant to your study. If you are not sure if a list item applies to your research, read the appropriate section before selecting a response.

Materials & experimental systems

n/a	Involved in the study	
☐	☒	Antibodies
☐	☒	Eukaryotic cell lines
☒	☐	Palaeontology and archaeology
☒	☐	Animals and other organisms
☐	☒	Clinical data
☒	☐	Dual use research of concern
☒	☐	Plants

Methods

n/a	Involved in the study	
☒	☐	ChIP-seq
☒	☐	Flow cytometry
☒	☐	MRI-based neuroimaging

Antibodies

Antibodies used	The manufacturer, catalog number, clone identifier and applied of all monoclonal and secondary antibodies used for Western Blot and Digi West can be found in the Supplementary Table 5 and the Supplementary File, respectively.
Validation	All antibodies are commercially available and were validated for Western Blot by the manufacturer. The specify of selected phopsho-antibodies for Western Blot was further verified experimentally (see Sup Figure 2). Due to the good comparability of WB and DigiWest (see Treindl et al., Nature Communication 2016), suitability for DigiWest can be assumed. Furthermore for DigiWest, each antibody is validated internally by testing its DigiWest assay performance on a variety of tissues and sample types. Only antibodies with satisfactory performance were used for this manuscript.

Eukaryotic cell lines

Policy information about cell lines and Sex and Gender in Research

Cell line source(s)	The NIH3T3 cell line was a kind gift by Wolfgang Neubert (Max Planck Institute for Biochemistry, Martinsried, Germany)
Authentication	The NIH3T3 cell line was authenticated by Mouse Short Tandem Repeat (STR) profile Report Cell Line Authentication Service by ATCC (Manassas, USA).
Mycoplasma contamination	Cell lines were tested negative for Mycoplasma contamination
Commonly misidentified lines (See ICLAC register)	No commonly misidentified cell lines was used in this study

Clinical data

Policy information about clinical studies

All manuscripts should comply with the ICMJE guidelines for publication of clinical research and a completed CONSORT checklist must be included with all submissions.

Clinical trial registration	No trial registration due to the retrospective nature of this study
Study protocol	No study protocol as this is a retrospective analysis of eight patients with FGFR2-driven CCA treated with lenvatinib
Data collection	No data collection as this is a retrospective analysis of eight patients with FGFR2-driven CCA treated with lenvatinib
Outcomes	As this is a retrospective analysis, no primary or secondary outcomes were pre-defined. Radiologic response was used as the primary outcome measure and assessed by an experienced radiologist based on principles of RECIST version 1.1. criteria.
